# Withaferin A Enhances Mitochondrial Biogenesis and BNIP3-Mediated Mitophagy to Promote Rapid Adaptation to Extreme Hypoxia

**DOI:** 10.3390/cells12010085

**Published:** 2022-12-25

**Authors:** Ruzhou Zhao, Yixin Xu, Xiaobo Wang, Xiang Zhou, Yanqi Liu, Shuai Jiang, Lin Zhang, Zhibin Yu

**Affiliations:** 1 Department of Aerospace Physiology, Air Force Medical University, 169# Changle West Road, Xi’an 710032, China; 2 Beijing Institute of Biotechnology, Academy of Military Medical Sciences (AMMS), Beijing 100071, China; 3 Department of Hepatobiliary Surgery, Affiliated Tumor Hospital of Guangxi Medical University, Nanning 530021, China

**Keywords:** withaferin A, PGC-1α, BNIP3, mitochondrial biogenesis, mitophagy

## Abstract

Rapid adaptation to extreme hypoxia is a challenging problem, and there is no effective scheme to achieve rapid adaptation to extreme hypoxia. In this study, we found that withaferin A (WA) can significantly reduce myocardial damage, maintain cardiac function, and improve survival in rats in extremely hypoxic environments. Mechanistically, WA protects against extreme hypoxia by affecting BCL2-interacting protein 3 (BNIP3)-mediated mitophagy and the peroxisome proliferator-activated receptor γ coactivator 1α (PGC-1α)-mediated mitochondrial biogenesis pathway among mitochondrial quality control mechanisms. On the one hand, enhanced mitophagy eliminates hypoxia-damaged mitochondria and prevents the induction of apoptosis; on the other hand, enhanced mitochondrial biogenesis can supplement functional mitochondria and maintain mitochondrial respiration to ensure mitochondrial ATP production under acute extreme hypoxia. Our study shows that WA can be used as an effective drug to improve tolerance to extreme hypoxia.

## 1. Introduction

Acute hypoxic injury is a problem that scientists have been trying to solve. As altitude increases, the partial pressure of oxygen in the body gradually decreases, which may induce acute altitude sickness, high-altitude pulmonary edema, high-altitude cerebral edema, and even life-threatening conditions [1,2]. Recently, people have had an increasing need to enter extremely high-altitude areas (>7620 m), for example, for performing tasks, traveling, and climbing Mount Everest; thus, rapid adaptation to extreme hypoxia has attracted increasing attention. At an altitude of 7620 m (25,000 ft), the ambient oxygen partial pressure is 60 mmHg, and the alveolar oxygen partial pressure attains the critical value of 30 mmHg. Staying at this altitude for even a few minutes causes the alveolar oxygen partial pressure to drop below 30 mmHg and triggers loss of consciousness. Therefore, the area above the altitude of 7620 m has been called the “death zone” [1,3], and even after acclimation, the human body cannot stay at this altitude for a long time. Few studies have addressed the rapid entry of the body into an extremely hypoxic environment such as that found above 7620 m, and no effective regimen to achieve rapid adaptation to extreme hypoxia has been reported.

Withaferin A (WA), a chemical component extracted from the popular Indian herb *Withania somnifera*, can block the activity of nuclear factor-κB (NF-κB) and inhibit angiogenesis and cell proliferation [4]. WA has anti-inflammatory, antidiabetic, and antitumor effects, but the efficacy of WA in protection against hypoxia has not been reported. Promisingly, our study showed that WA pretreatment significantly increased the 24 h survival rate of Sprague-Dawley (SD) rats at 7620 m, suggesting that WA plays an important role in enhancing the rapid adaptability to extreme hypoxia. In this study, we confirmed that WA plays a protective role against extreme hypoxia by affecting mitochondrial quality control mechanisms.

## 2. Materials and Methods

### 2.1. Animals and Reagents

Adult male SD rats (10–12 weeks, >300 g) were used in this experiment. Rats were randomly divided into four groups, as follows: Con group, normal rats at the baseline altitude of 412 m; H group, rats exposed to a simulated altitude of 7620 m for 24 h; W group, rats given intraperitoneal injections of 2 mg/kg WA for 7 days at the baseline altitude of 412 m; WH group, rats given intraperitoneal injections of 2 mg/kg WA for 7 days and then exposed to 7620 m for 24 h. The rats in Con and H group were injected with vehicle before the corresponding treatment. WA (5119-48-2, IC-0201518) was purchased from InCellGene and was solubilized in 10% DMSO and 90% corn oil.

### 2.2. Simulation of Hypobaric Hypoxia

The hypobaric hypoxic environment was created in a hypobaric chamber system as previously described [5]. After the height value was input into the control system, the hypobaric hypoxic environment was created in the cabin via the vacuum pump. The cabin was ventilated, and the speeds of ascent and descent were controlled at 10 m/s. The temperature and humidity of cabins were maintained at 25 ± 5 °C and 50 ± 5%. The survival of rats was recorded during 24 h hypoxia.

### 2.3. Hematoxylin-Eosin (HE) Staining and Reactive Oxygen Species (ROS)/Adenosine Triphosphate (ATP) Detection in Cardiac Tissue

Rats were anesthetized with 1% pentobarbital sodium, and the cardiac tissue was removed, immediately frozen in liquid nitrogen, and stored at −80 °C for formalin-fixed section preparation, Western blot analysis, quantitative PCR (qPCR), and tissue ROS and ATP measurement. For HE staining, the cardiac tissue sections were stained with hematoxylin and eosin, and images were acquired by photomicroscopy.

The myocardial ROS level was determined by DHE staining (S0063, Beyotime Biotechnology, China). The freshly frozen myocardial tissue sections were immediately stained with DHE reaction solution. The sections were detected at 535 nm excitation, and the fluorescence intensity was analyzed using NIH ImageJ software.

The myocardial ATP content was determined by ATP Assay Kit (S0026, Beyotime Biotechnology, China) according to the instructions. Briefly, the myocardium was split into homogenates, and the supernatant was removed after being centrifuged, and then the luminescence value of the sample was detected using the Tecan microplate reader after the ATP detection solution was added.

### 2.4. Echocardiography

Echocardiography was performed as previously described [6]. In brief, rats were anesthetized with 3% isoflurane, and the prethoracic area was exposed by depilation. Then, the heart was scanned, and M-mode images were acquired. The following parameters were recorded: heart rate (HR), left ventricular internal dimension at end-systole (LVIDs), left ventricular internal dimension at end-diastole (LVIDd), left ventricular end-diastolic volume (LVEDV), and left ventricular end-systolic volume (LVESV). Left ventricular ejection fraction (LVEF) and left ventricular fractional shortening (LVFS) were calculated as LVEF = [(LVEDV − LVESV)/(LVEDV)] and LVFS = [(LVIDd − LVIDs)/(LVIDd)]. In addition, the E and A peak velocities were recorded, and E/A ratio was calculated to reflect the diastolic function.

### 2.5. Cell Culture and Treatments

The human cardiomyocyte cell line AC16 was used in this experiment. Cells in the control (Con) group were cultured in DMEM supplemented with 10% FBS in 5% CO_2_ at 37 °C. Cells in the W group were treated with 0.5 μmol/L WA for 48 h. For acute hypoxia simulation, cells in the Con and W group were exposed to 1% O_2_ for 24 h (“H” group and “WH” group, respectively) using a tri-gas incubator (Heal Force-HF100, Shanghai, China).

For PGC-1α transduction experiment, cells in culture were transfected with empty vector adenovirus (Ad-Con) or PGC-1α expression adenovirus (Ad-PGC1α, Hanbio, Shanghai, China). At 36 h after transfection, cells were treated with hypoxia as above.

### 2.6. Western Blot Analysis

As described previously [7], protein was extracted from myocardial tissue or cells after different treatments, and the protein concentration was measured by the BCA method [7]. The protein samples were mixed with loading buffer and heated for denaturation. After electrophoresis and membrane transfer in an XCell SureLock Mini-Cell apparatus (Invitrogen, Carlsbad, CA, USA), proteins were transferred to a PVDF membrane. After blocking with 5% BSA, the membrane was incubated with the primary antibody and horseradish peroxidase (HRP)-conjugated secondary antibody. Bands were detected using ECL reagents (Millipore, Burlington, MA, USA). The gray values of the target protein bands were measured with NIH ImageJ software. The antibodies used in this experiment are described in the Supplemental Material.

### 2.7. qRT-PCR

For measurement of mRNA levels, total RNA from cardiac tissue or AC16 cells was extracted using a TRIzol kit (Invitrogen, CA, USA) according to the protocol. One microgram of total RNA was reverse transcribed to cDNA using a PrimeScript RT Reagent Kit (Takara, Tokyo, Japan). RT-PCR analysis was performed using a CFX96 (Bio-Rad, CA, USA) instrument and SYBR Premix Ex Taq (Takara, Tokyo, Japan) following the instructions. The housekeeping gene β-actin was used as the internal reference. The 2^−ΔΔCt^ method was used to determine the relative mRNA levels of target genes.

To determine the mitochondrial DNA (mtDNA) content, the level of the mitochondria-encoded gene NADH dehydrogenase subunit 1 (ND-1) was normalized to that of the nuclear housekeeping gene β-actin to reflect the number of mitochondria.

The primers specific for peroxisome proliferator-activated receptor γ coactivator 1α (PGC-1α), nuclear factor erythroid 2-related factor 2 (NRF2), NRF1, mitochondrial transcription factor A (TFAM), BCL2-interacting protein 3 (BNIP3), ND-1, and β-actin mRNA used in this experiment are listed in the Supplemental Material.

### 2.8. Transmission Electron Microscopy (TEM)

For tissue TEM, fresh cardiac tissues were immediately fixed with 4% glutaraldehyde for 24 h and were then trimmed into 0.1 × 0.1 × 0.1 cm^3^ blocks for further fixation with 1% osmium tetroxide in deionized water. For TEM, the adherent cells were digested, washed twice with PBS, and fixed with 4% glutaraldehyde for 24 h. Subsequent steps were performed as previously described [8]. Electron micrographs were acquired using a transmission electron microscope (HT7800 series, Hitachi, Tokyo, Japan) at 80 kV. The morphology of myocardial fibers, mitochondria, and autophagosomes was observed. At least five visual fields per sample were randomly selected to analyze the number of mitochondria.

### 2.9. Analysis of Apoptosis and Measurement of the Mitochondrial Membrane Potential (MMP)

An apoptosis/necrosis detection kit (ab176749, Abcam, MA, USA) was used to simultaneously monitor apoptotic (green), necrotic (red), and healthy (blue) cells in strict accordance with the manufacturer’s instructions. The stained samples were analyzed by flow cytometry (Beckman Coulter, Brea, CA, USA) and fluorescence microscopy (LSM800, Carl Zeiss, Germany).

The MMP was measured using a JC-1 mitochondrial membrane potential assay kit (C2006, Beyotime Biotechnology, Shanghai, China). Cells were incubated with JC-1 solution at 37 °C for 20 min, and imaging was then performed using a high-content imaging system (Harmony, PerkinElmer, Rodgau, Germany). The ratio of red to green fluorescence intensity was used to determine the MMP.

### 2.10. Measurement of ROS with RoGFP Sensors in Subcellular Compartments

Cyto-RoGFP (Addgene, #49435) and IMS-RoGFP (Addgene, #49436) were employed to determine ROS levels in the cytoplasm and mitochondrial intermembrane space (IMS). The principle of RoGFP sensors was previously described [9,10]. In RoGFP-transfected cells, blue fluorescence excited at 405 nm reflects the oxidation state, while green fluorescence excited at 488 nm reflects the reduction state. The ratio of the two fluorescence intensities indicates the intracellular ROS level. The fluorescent images were acquired at 36 h post-transfection as the oxidative stress status before hypoxia. Then, the cells were treated with 1% O_2_ for 24 h followed by images capture to indicate the oxidative stress status after hypoxia. The fluorescent images were taken using high-content imaging and analysis software (Perkin Elmer, Woodbridge, ON, USA).

### 2.11. Measurement of Mitochondrial ATP with mitGO-ATeam2

A FRET-based fluorescent ATP probe, mitGO-ATeam2 (a gift from Hiromi Imamura, Kyoto University), was used to monitor the mitochondrial ATP level in living cells. The probe was previously described [10,11]. Cardiomyocytes were transfected with the pcDNA-mitGo-ATeam2 plasmid using Lipo3000 (Invitrogen, CA, USA). The mitGO-ATeam2 sensor was excited at 488 nm, and emission was measured at 510 nm and 560 nm. The ratio of fluorescence emission intensity at 560 nm to that at 510 nm indicates the ATP level in cells. The fluorescent images were acquired similarly as described in Section 2.10.

### 2.12. Measurement of the Mitochondrial Oxygen Consumption Rate (OCR)

An XF24 extracellular flux analyzer (Agilent Seahorse Bioscience, Santa Clara, CA, USA) was employed to measure the OCR, as previously described [12]. Analysis was carried out according to the manufacturer’s instructions. Values were normalized to the intracellular protein concentration and are presented as pmol/min/μg protein. The OCR parameters, including basal respiration, ATP-linked respiration, maximal respiration rate, and spare respiratory capacity, were calculated and analyzed.

### 2.13. Statistical Analysis

All experiments were independently repeated at least 3 times, and results are presented as the means ± SEMs values. For comparisons among multiple groups, two-way ANOVA followed by Tukey’s multiple comparison test was used. *p* < 0.05 was considered to indicate a significant difference. Statistical analysis and graphs were achieved using GraphPad Prism 8.0 (GraphPad Software, San Diego, CA, USA).

## 3. Results

### 3.1. WA Attenuated Injury Caused by Extreme Hypoxia and Increased the Survival Rate of Rats under Extreme Hypoxia

Figure S1 and Table S1 show the survival rate of SD rats at the simulated altitude of 7620 m within 24 h and after 24 h, respectively. More than half of the rats died within 6 h after attaining the altitude of 7620 m (Figure S1). The 24 h survival rate of normal SD rats in the Con group at 7620 m was 9.5%. In contrast, the survival rate of SD rats in the WA pretreatment group was significantly higher, i.e., 85.7% (Table S1).

**Figure 1:**
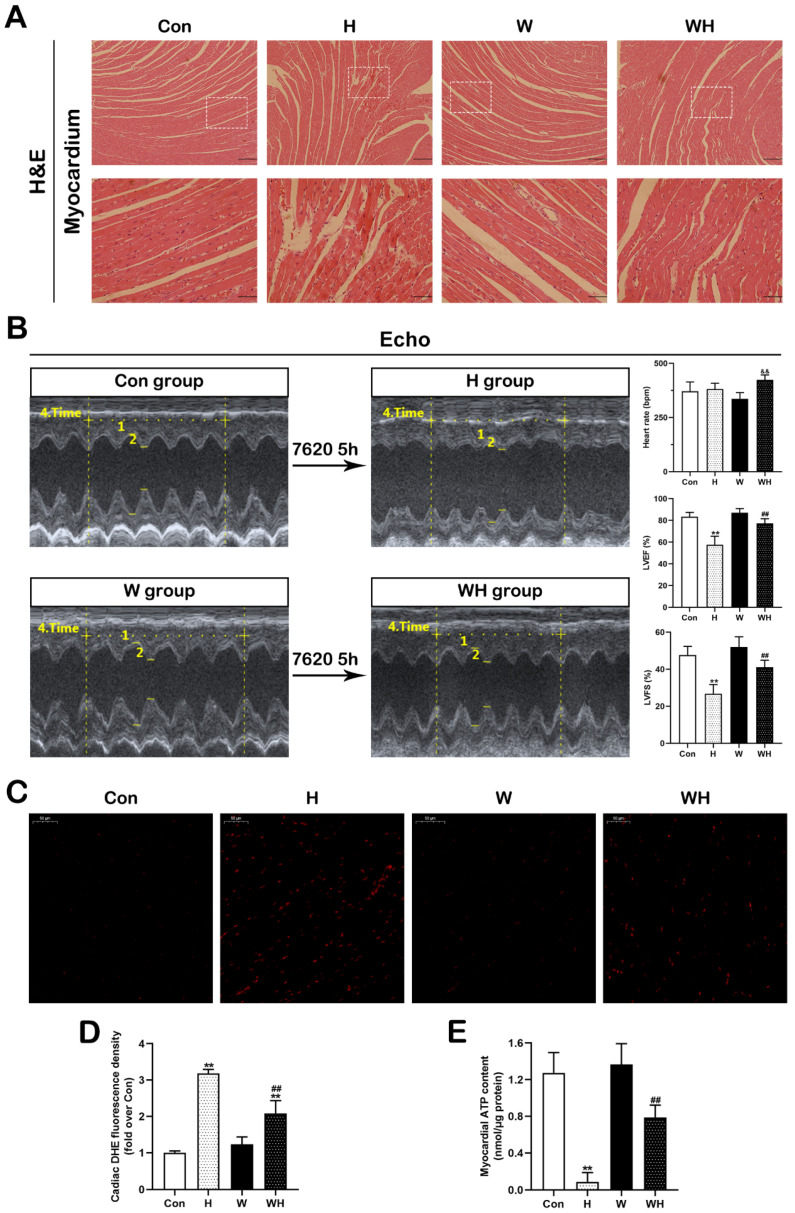
WA attenuated myocardial injury and maintained cardiac function during acute extreme hypoxia. (**A**) HE staining of myocardial tissue in SD rats. Scale bars = 200 μm and 50 μm (enlarged image). (**B**) Typical image of myocardial echocardiography and quantitative analysis results for HR, LVEF, and LVFS. Echocardiography was performed on SD rats in the following stages: before hypoxia treatment and after maintenance at 7620 m for 5 h (Con–H groups and W–WH groups, respectively). (**C**,**D**) Representative fluorescence images of DHE staining in the different treatment groups (scale bar = 50 μm) (**C**) and statistical results of the mean DHE fluorescence intensity (**D**). (**E**) ATP content in myocardial tissue. The values are presented as the means ± SEMs. *n* = 5 rats/group. ** *p* < 0.01 vs. the Con group. ^##^ *p* < 0.01 vs. the H group. ^&&^ *p* < 0.01 vs. the W group.

Myocardial HE staining showed that the myocardial morphology in the W group was not significantly different from that in the Con group, and both showed normal myocardial fiber morphology. Compared with the Con group, the H group exhibited severe myocardial tissue injury, as evidenced by the disordered myocardial fiber arrangement and abundant fiber dissolution and breakage, while the rats in the WH group had significantly less myocardial tissue damage than those in the H group. Although fiber disorganization was observed, no myocardial fiber breakage was found in the rats in the WH group (Figure 1A).

After rats had been maintained at the altitude of 7620 m for 5 h, echocardiography was used to evaluate the cardiac function of the surviving rats. The LVEF of SD rats in the Con group decreased from 83.5% to 57.5%, and the LVFS decreased from 47.6% to 26.7%, while the mean values of LVEF and LVFS in the WH group were 77.2% and 41.1%, respectively. The E/A ratio was significantly improved in the WH group. These results indicated that the cardiac function of rats in the WH group was significantly better than that of rats in the H group (Figure 1B and Figure S2). ROS and ATP measurement in myocardial tissues showed that the H group had the most significant increase in myocardial ROS and the lowest ATP concentration; the WH group had less ROS generation and a higher ATP concentration than the H group (Figure 1C–E).

### 3.2. WA Enhanced Mitochondrial Biogenesis and BNIP3-Mediated Mitophagy in Myocardial Tissues

We first measured the expression of proteins involved in mitochondrial fission (dynamin-related protein 1 (DRP1) and mitochondrial fission protein 1 (FIS1)) and fusion (optic atrophy protein 1 (OPA1), mitofusin 1 (MFN1), and mitofusin 2 (MFN2)). No significant changes in mitochondrial fission or fusion protein expression were observed in any group, suggesting that WA did not affect mitochondrial fission or fusion activity in myocardial tissues (Figure S3).

**Figure 2:**
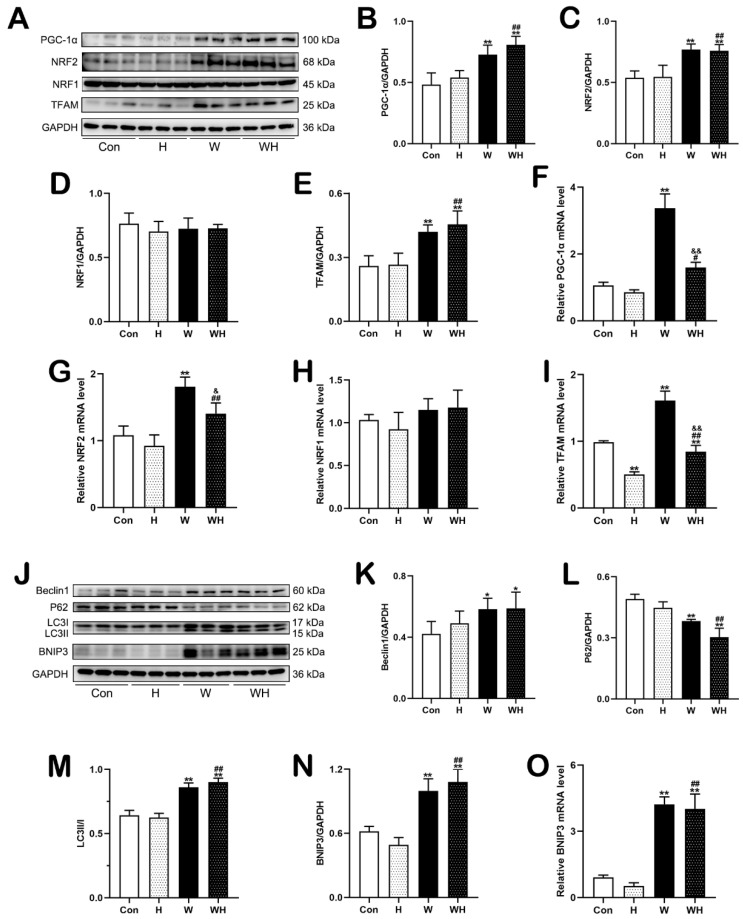
WA enhanced mitochondrial biogenesis and BNIP3-mediated mitophagy in myocardial tissue. (**A**–**E**) Representative immunoblots (**A**) and statistical graphs of the protein expression of PGC-1α (**B**), NRF2 (**C**), NRF1 (**D**), and TFAM (**E**). (**F**–**I**) Relative mRNA levels of genes involved in mitochondrial biogenesis. (**J**–**N**) Representative immunoblots (**J**) and statistical graphs of the protein expression of Beclin1 (**K**), P62 (**L**), LC3 (**M**), and BNIP3 (**N**). (**O**) Relative mRNA level of BNIP3. The values are presented as the means ± SEMs (*n* = 5 animals/group). * *p* < 0.05 or ** *p* < 0.01 vs. the Con group. ^#^ *p* < 0.05 or ^##^ *p* < 0.01 vs. the H group. ^&^ *p* < 0.05, ^&&^ *p* < 0.01 vs. the W group.

Next, we determined the expression levels of mitochondrial biogenesis-related proteins in myocardial tissues (Figure 2A). The myocardial protein levels of PGC-1α, NRF2, and TFAM in rats in the H group were not significantly different from those in rats in the Con group. WA treatment significantly increased the protein expression of PGC-1α, NRF2, and TFAM in myocardial tissues in the W group and WH group. In addition, the protein levels of PGC-1α, NRF2, and TFAM in myocardial tissues were higher in the WH group than in the H group (Figure 2B,C,E). There was no significant difference in the NRF1 protein level between the groups (Figure 2D). Then, we measured the levels of transcripts encoding mitochondrial biogenesis-related proteins and found that the levels of PGC-1α, NRF2, and TFAM mRNA in myocardial tissues were significantly higher in the W group than in the Con group. The TFAM mRNA level in myocardial tissues was lower in the H group than in the Con group. Although the mRNA levels of PGC-1α and NRF2 in the H group were not significantly different from those in the Con group, they did show a decreasing trend. The mRNA levels of PGC-1α, NRF2, and TFAM in the WH group were significantly increased compared with those in the H group but were lower than those in the W group. The mRNA level of NRF1 did not differ significantly among the groups (Figure 2F–I).

Western blot analysis of autophagy-related proteins (Figure 2J) showed that the protein levels of beclin1, p62, and LC3II/I in myocardial tissues of the H group did not change significantly. However, the levels of beclin1 and LC3II/I in the W and WH groups were significantly higher than those in the Con group, while the p62 level was significantly decreased in the W and WH groups. The changes in protein levels were the most significant in the WH group, and the levels obviously differed from those in the H group. The protein level of the mitophagy-related protein BNIP3 did not change significantly in the H group but increased markedly in the W and WH groups and was significantly higher in the WH group than in the H group (Figure 2K–N). qPCR showed that the BNIP3 mRNA level in the W and WH groups was significantly higher than that in the Con group and was significantly higher in the WH group than in the H group (Figure 2O).

**Figure 3:**
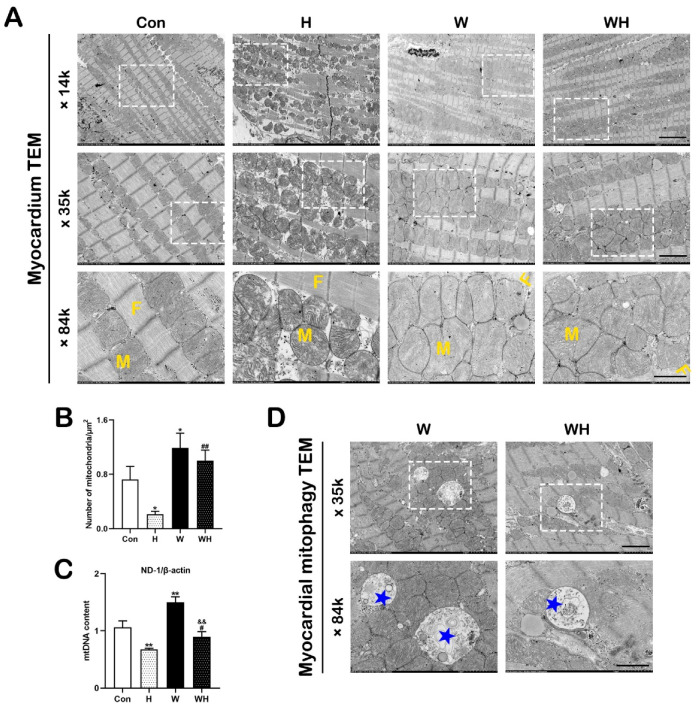
WA increased the mitochondrial number and enhanced autophagic flux in myocardial tissue. (**A**) Representative electron micrograph showing myocardial fibers and mitochondria. The dashed boxes correspond to the enlarged regions. M, mitochondria; F, myocardial fibers. Scale bars = 5 μm (14 K ×), 2 μm (35 K ×), 1 μm (84 K ×). (**B**) Number of mitochondria per μm^2^. Mitochondria were counted in five randomly selected TEM visual fields per group. (**C**) mtDNA copy numbers in the myocardium, as evaluated by qPCR. (**D**) Representative electron micrograph showing autophagosomes in the myocardium in the W and WH groups, as indicated by the blue pentagrams. The values are presented as the means ± SEMs (*n* = 5 animals/group). * *p* < 0.05 or ** *p* < 0.01 vs. the Con group. ^#^ *p* < 0.05 or ^##^ *p* < 0.01 vs. the H group. ^&&^ *p* < 0.01 vs. the W group.

We observed mitochondria and myocardial fibers by TEM and statistically analyzed the number of mitochondria. In the Con group, the myocardial fibers were arranged in an orderly manner, and mitochondria were arranged in register with the muscle fibers, with obvious mitochondrial cristae. In rats in the H group, the myocardial fibers were disorganized and exhibited dissolution and even breakage, and the mitochondria were swollen, with ruptured cristae. The damage to myocardial fibers and mitochondria in the WH group was significantly attenuated compared with that in the H group, and no significant muscle fiber rupture or mitochondrial swelling was observed (Figure 3A). The number of mitochondria in random TEM fields of view was statistically analyzed. The number of mitochondria in myocardial tissues in the W and WH groups was significantly higher than that in the Con group, while the number of myocardial mitochondria in the H group was significantly lower than that in the Con and WH groups (Figure 3B). The number of mitochondrial gene replications was detected by qPCR. The mtDNA content was significantly lower in the H group but significantly higher in the W group than in the Con group. The myocardial mtDNA content in the WH group was significantly lower than that in the W group but still significantly higher than that in the H group (Figure 3C).

Finally, TEM was used to observe autophagy in myocardial tissues in each group. Autophagosomes were observed in the myocardial tissues of the W and WH groups, encapsulating glycogen, mitochondria, and other cellular content. Typical images are shown in Figure 3D.

### 3.3. WA Attenuated Acute Hypoxic Injury in Cardiomyocytes

Through a CCK-8 assay, we confirmed that treatment of cardiomyocytes with 0.5 μmol/L WA for 48 h did not cause toxic damage to the cardiomyocytes (Figure S4). Therefore, 0.5 μmol/L treatment for 48 h was used as the protocol for in vitro experiments.

**Figure 4:**
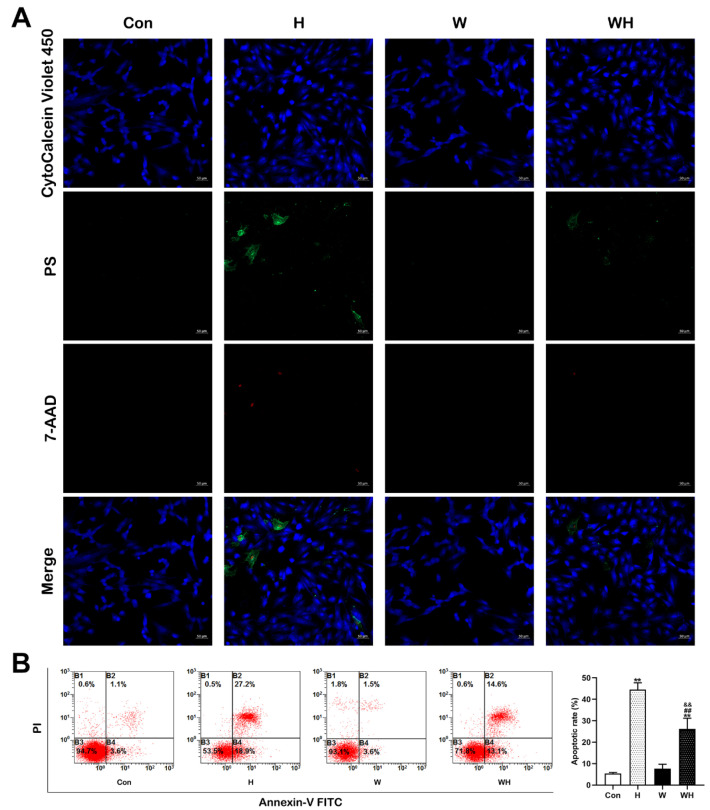
WA attenuated cardiomyocyte apoptosis after acute hypoxia. (**A**) Immunofluorescence analysis of normal (stained blue), apoptotic (stained green), and necrotic (stained red) cardiomyocytes. Scale bars = 50 μm. (**B**) Flow cytometric analysis of total apoptotic cells (bottom right quadrant, viable apoptotic cells; upper right quadrant, nonviable apoptotic cells) by Annexin V and PI double staining in cardiomyocytes. The data show the mean ± SEM of three independent experiments. ** *p* < 0.01 vs. the Con group. ^##^ *p* < 0.01 vs. the H group. ^&&^ *p* < 0.01 vs. the W group.

Simultaneous immunofluorescence staining of live, apoptotic, and necrotic cardiomyocytes was performed using an apoptosis/necrosis detection kit. As shown in Figure 4A, live cells were stained violet blue, apoptotic cells were stained green by the phosphatidylserine indicator, and necrotic cells were stained red by 7-AAD. Under a fluorescence microscope, the number of green- and red-stained cardiomyocytes in the H group was the highest among all groups. Although green- and red-stained cells were observed, the number was significantly lower than that in the H group.

Moreover, flow cytometric analysis was performed to detect apoptotic cardiomyocytes in each group. After 1% O_2_ hypoxia treatment for 24 h, the apoptosis rate of cardiomyocytes in the H group was significantly increased, and the apoptosis rate of cardiomyocytes in the WH group was significantly lower than that in the H group, although it was higher than that in either the Con group or W group (Figure 4B).

### 3.4. WA Enhanced Mitochondrial Biogenesis and BNIP3-Mediated Mitophagy in Cardiomyocytes

**Figure 5:**
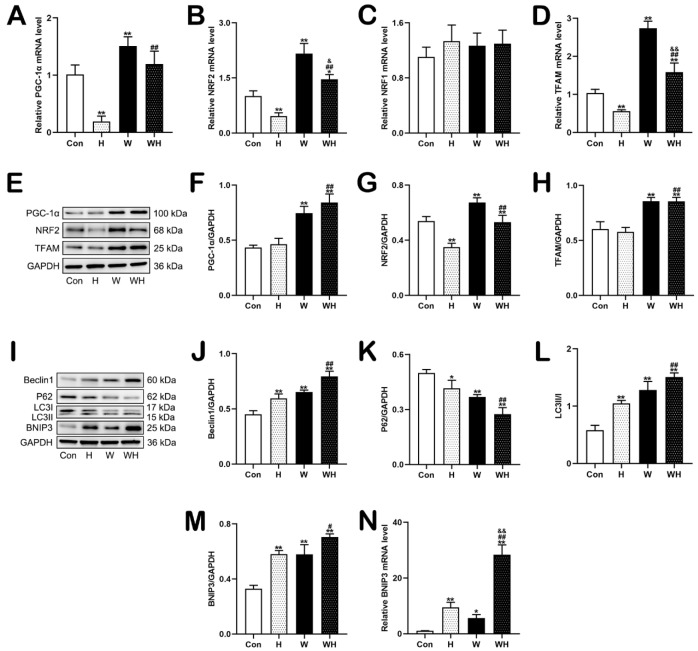
WA enhanced mitochondrial biogenesis and BNIP3-mediated mitophagy in cardiomyocytes. (**A**–**D**) Relative mRNA expression of PGC-1α (**A**), NRF2 (**B**), NRF1 (**C**), and TFAM (**D**). (**E**) Representative immunoblots of proteins involved in mitochondrial biogenesis. (**F**–**H**) Quantification of mitochondrial biogenesis-related protein (PGC-1α, NRF2, and TFAM) expression in cardiomyocytes. (**I**) Representative immunoblots of autophagy-related proteins (Beclin1, P62, LC3) and a mitophagy-related protein (BNIP3) in cardiomyocytes. (**J**–**M**) Statistical graphs showing relative expression of the indicated proteins. (**N**) Relative mRNA level of BNIP3 in cardiomyocytes. The values are presented as the mean ± SEM of three independent experiments (*n* = 3). * *p* < 0.05 or ** *p* < 0.01 vs. the Con group. ^#^ *p* < 0.05 or ^##^ *p* < 0.01 vs. the H group. ^&^ *p* < 0.05 or ^&&^ *p* < 0.01 vs. the W group.

qPCR showed that the levels of PGC-1α, NRF2, and TFAM mRNA in cardiomyocytes in the H group were significantly decreased after 24 h of acute hypoxia. The levels of these mRNAs in cardiomyocytes were significantly higher in the W group than in the Con group. After 24 h of acute hypoxia, the cardiomyocyte levels of NRF2 and TFAM mRNA in the WH group were significantly decreased compared to those in the W group but were significantly higher than those in the H group, which were even higher than those in the Con group (Figure 5A,B,D). The NRF1 mRNA level did not differ significantly among the groups (Figure 5C). Western blot analysis further confirmed that the PGC-1α, NRF2, and TFAM protein levels were significantly increased in cardiomyocytes in the W and WH groups and that the levels of these proteins in the WH group were significantly higher than those in the H group. In addition, the NRF2 protein level in cardiomyocytes was significantly lower in the H group than in the Con group (Figure 5E–H).

To further determine the role of elevated PGC-1α in acute hypoxic cells, PGC-1α expressing adenovirus was employed. Electron microscopy results showed that overexpression of PGC-1α significantly increased the number of mitochondria in cardiomyocytes and significantly reduced the number of damaged mitochondria after acute hypoxia (Figure S5A–C). In addition, overexpression of PGC-1α obviously improved mitochondrial respiratory function of cardiomyocytes under acute hypoxic environment and especially enhanced the basal respiration, ATP-production-linked respiration, and maximal respiration (Figure S5D–E).

The levels of autophagy-related proteins and the BNIP3 protein were also determined by Western blot analysis. Compared with the Con group, the H, W, and WH groups had significantly increased expression of beclin1, decreased expression of p62, an increased LC3II/I ratio, and increased expression of BNIP3 in cardiomyocytes. The change trends in these proteins were the most obvious in the WH group, as their levels were significantly different from those in the H group. In addition, the level of BNIP3 mRNA in the H, W, and WH groups was significantly higher than that in the Con group, and the increase in the BNIP3 mRNA level was the most significant in the WH group, in which it was significantly higher than that in the H and W groups.

**Figure 6:**
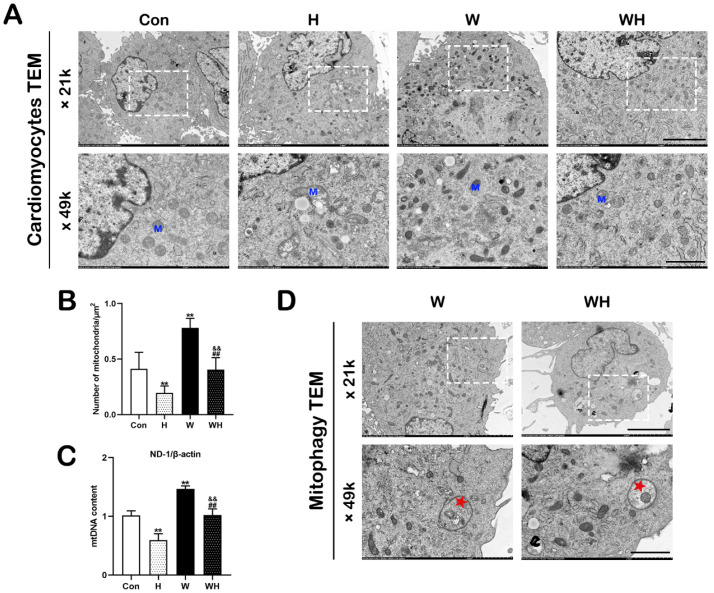
WA increased the mitochondrial number and enhanced autophagic flux in cardiomyocytes. (**A**) Representative electron micrograph of cardiomyocytes from each group. The dashed boxes correspond to the enlarged regions. M, mitochondria. Scale bars = 5 μm (21 K×), 2 μm (49 K×). (**B**) Number of mitochondria per μm^2^. Mitochondria were counted in six randomly selected TEM visual fields per group. (**C**) mtDNA content in cardiomyocytes, as evaluated by qPCR. (**D**) Representative electron micrograph showing autophagosomes in cardiomyocytes in the W and WH groups, as indicated by the red pentagrams. The values are presented as the mean ± SEM of three independent experiments (*n* = 3). ** *p* < 0.01 vs. the Con group. ^##^ *p* < 0.01 vs. the H group. ^&&^ *p* < 0.01 vs. the W group.

Cellular mitochondrial morphology and autophagy were observed by TEM. A typical graph is shown in Figure 6A. In the H group, mitochondria were severely damaged, mitochondrial cristae disappeared, and many mitochondria were swollen and ruptured. Mitochondrial damage was significantly alleviated in the WH group. Moreover, statistical analysis of random TEM fields of view showed that acute hypoxia resulted in a decrease in the number of mitochondria in cells in the H group compared to that in cells in the Con group. In contrast, the number of mitochondria in cardiomyocytes was significantly increased in the W group. Although the number of mitochondria was also reduced in the WH group after acute hypoxia, it was still significantly higher than that in the H group (Figure 6B). Furthermore, qPCR confirmed that the mtDNA copy number was significantly reduced in the H group but was significantly increased in the W group after WA treatment compared to that in the Con group. The mtDNA content in the WH group was significantly higher than that in the H group and lower than that in the W group (Figure 6C). In addition, autophagosomes, which encapsulated mitochondria and other cellular content, were obvious in the W and WH groups by TEM (Figure 6D).

### 3.5. WA Reduced ROS Production in Cardiomyocytes under Acute Hypoxia

**Figure 7:**
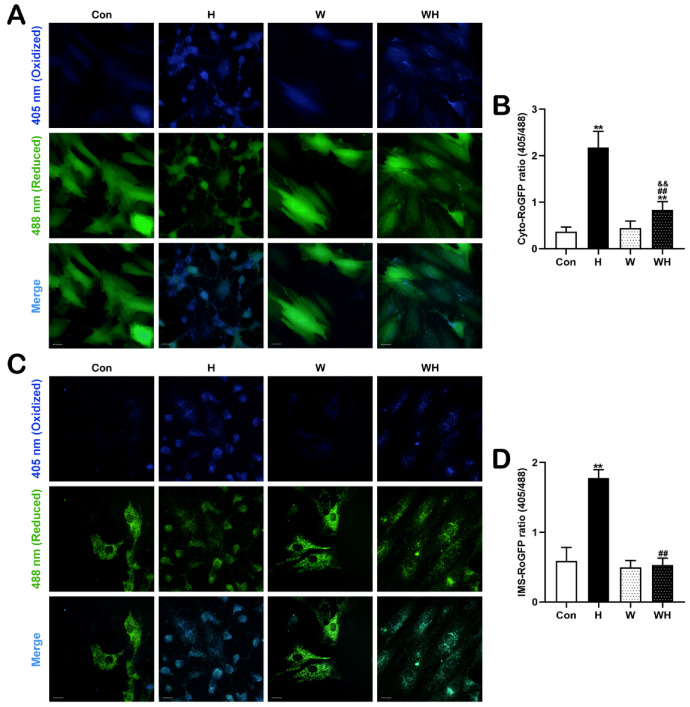
WA reduced ROS production in cardiomyocytes during acute hypoxia. (**A**) Representative immunofluorescence images of cardiomyocytes transfected with Cyto-RoGFP. Excitation at 405 nm and 488 nm indicates the oxidation and reduction states, respectively. (**B**) Quantitative analysis of cytosolic ROS in cardiomyocytes, as reflected by the ratio of the fluorescence intensity at 405 nm to that at 488 nm. Scale bars = 20 μm. (**C**) Immunofluorescence images of cardiomyocytes transfected with IMS-RoGFP: 405 nm, oxidation; 488 nm, reduction. (**D**) Statistical analysis of IMS ROS, as reflected by the ratio of the fluorescence intensity at 405 nm to that at 488 nm. Scale bars = 20 μm. The data are presented as the mean ± SEM values; *n* > 20 fields from three independent experiments. ** *p* < 0.01 vs. the Con group. ^##^ *p* < 0.01 vs. the H group. ^&&^ *p* < 0.01 vs. the W group.

Cyto-RoGFP and IMS-RoGFP were used to detect ROS in the cytoplasm and IMS of living cardiomyocytes, respectively. In Figure 7A,C, blue fluorescence and green fluorescence indicate the oxidation state and reduction state, respectively, of cardiomyocytes. In cardiomyocytes in the Con and W groups, the blue fluorescence of Cyto-RoGFP and IMS-RoGFP was relatively weak. After acute hypoxia, the blue fluorescence of Cyto-RoGFP and IMS-RoGFP in cardiomyocytes in the H group was significantly enhanced, and green fluorescence was significantly attenuated. The blue and green fluorescence intensities in the WH group were different from those in the Con group, but the changes in the WH group were not as significant as those in the H group.

The ratio of blue to green fluorescence intensity can indicate the ROS content. Statistical analysis showed that WA had no significant effect on ROS in the cytoplasm or IMS of cardiomyocytes. The ROS level in the cytoplasm was significantly higher in both the H group and the WH group than in the Con group, but the increase was the most significant in the H group (Figure 7B). Similarly, compared with that in the Con group, the IMS ROS level was significantly increased in the H group, but the increase in the WH group was not significant; indeed, the IMS ROS level in the WH group was significantly lower than that in the H group (Figure 7D).

### 3.6. WA Maintained the MMP and Mitochondrial Function in Cardiomyocytes under Acute Hypoxia

**Figure 8:**
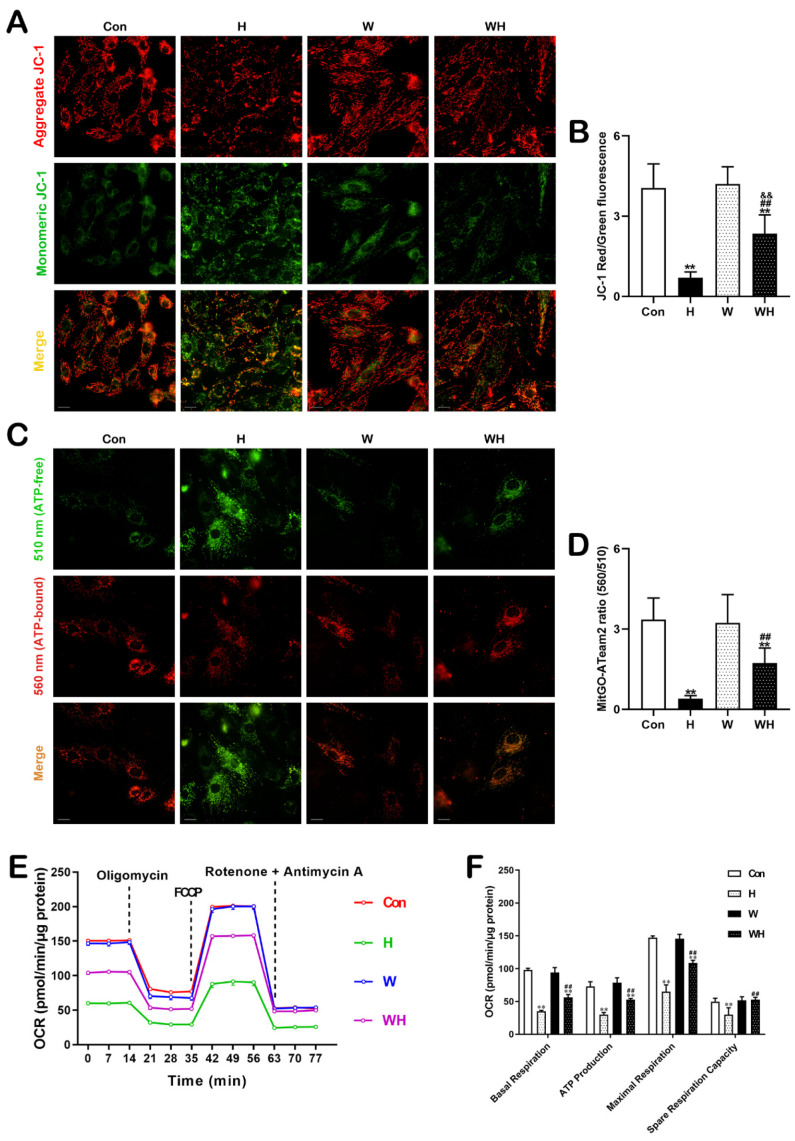
WA maintained the MMP and restored ATP production in the mitochondria of cardiomyocytes. (**A**) Representative immunofluorescence images of JC-1 staining; red fluorescence indicates JC-1 aggregates, and green fluorescence indicates monomeric JC-1. Scale bars = 20 μm. (**B**) Statistical analysis of the MMP, presented as the ratio of aggregated to monomeric JC-1. (**C**) Representative immunofluorescence images of cardiomyocytes transfected with MitGO-ATeam2. Scale bars = 20 μm. (**D**) Quantitative analysis of mitochondrial ATP levels, as indicated by the ratio of emission at 560 nm to that at 510 nm. (**E**) Line graph and statistical analysis of OCR. (**F**) Quantification of basal respiration, ATP production, maximal respiration, and spare respiration. The data are presented as the mean ± SEM values. In (**A**) and (**C**), *n* > 20 fields from three independent experiments; in (**E**), *n* = 5. ** *p* < 0.01 vs. the Con group. ^##^ *p* < 0.01 vs. the H group. ^&&^ *p* < 0.01 vs. the W group.

The MMP in cardiomyocytes was detected by JC-1 staining. As shown in Figure 8A, the changes in the fluorescence of the cells in the H group were the most significant, with red fluorescence decreasing and green fluorescence increasing, while the changes in the WH group were not significant. The ratio of red to green fluorescence intensity reflects the MMP. Compared with cardiomyocytes in the Con group, cardiomyocytes in the H and WH groups had a significantly lower MMP, but the reduction in the H group was the most significant, and the MMP was significantly lower than that in the WH group. In addition, the MMP in cardiomyocytes was reduced in the WH group compared to the W group (Figure 8B).

The mitochondrial ATP content was quantitatively assessed using the MitGO-ATeam2 plasmid. Typical plots are shown in Figure 8C. After acute hypoxia, the green fluorescence released at 510 nm by cardiomyocytes was significantly increased, while the red fluorescence released at 560 nm was attenuated. After WA pretreatment, this change was not significant in cardiomyocytes in the WH group. The ratio of the intensity of fluorescence released at 560 nm to the intensity of fluorescence released at 510 nm reflects the intracellular mitochondrial ATP content. The statistical results showed that the mitochondrial ATP content in cardiomyocytes was lower in the H group and the WH group than in the Con group, but the ATP reduction was the most significant in the H group, in which the ATP content was obviously lower than that in the WH group (Figure 8D).

Mitochondrial respiration was detected by measuring the OCR using the *Seahorse XF24* analyzer (Figure 8E). Acute hypoxia severely decreased the mitochondrial respiratory capacity, as indicated by the significant reductions in basal respiration, ATP-linked respiration, maximal respiration rate, and spare respiratory capacity in cardiomyocytes in the H group. Mitochondrial respiration in cardiomyocytes pretreated with WA was not significantly different from that in cardiomyocytes in the Con group. After acute hypoxia, the mitochondrial respiratory capacity in the WH group was slightly impaired, and the OCR was significantly higher in the WH group than in the H group (Figure 8F).

## 4. Discussion

Most studies on hypoxia adaptation have focused on rapid adaptation or acclimatization at mid–high altitudes (3000–5000 m). Few studies have addressed rapid adaptation to extreme hypoxia at altitudes above 7620 m. Our study, for the first time, showed that WA, which exerts anti-inflammatory and anticancer effects, can effectively promote rapid adaptation to extreme hypoxia. WA significantly increased the ability of SD rats to adapt to acute extreme hypoxia and increased the survival rate of SD rats after 24 h in an extremely hypoxic environment (at an altitude of 7620 m). Mechanistically, WA may play a protective role against extreme hypoxia by regulating cellular mitochondrial quality control mechanisms. With SD rats as the study’s subjects, we found that WA pretreatment significantly reduced myocardial tissue injury caused by extreme hypoxia, ensured the ATP supply to the heart, and improved cardiac function in the extremely hypoxic environment. By a combination of animal and cellular experiments, we confirmed that WA pretreatment impacted mitochondrial quality control mechanisms, which significantly enhanced mitochondrial biogenesis and BNIP3-mediated mitophagy in the myocardium and cardiomyocytes. The enhanced mitophagy could clear dysfunctional mitochondria damaged by hypoxia to prevent mitochondria-induced cell death, and the enhanced mitochondrial biogenesis could supply cells with an appropriate number of functioning mitochondria under extreme hypoxia. These two pathways, mitophagy and mitochondrial biogenesis, were complementary and maintained mitochondrial respiration to ensure the cellular ATP supply under extreme hypoxia. In summary, our study demonstrated for the first time that WA plays an important role in improving the body’s ability to rapidly adapt to extremely hypoxic environments. Regulation of myocardial mitochondrial quality control mechanisms—that is, upregulation of mitochondrial biogenesis and BNIP3-mediated mitophagy—may be an important mechanism by which WA exerts its anti-hypoxic effects.

Altitude is generally divided into three zones: 1500–3500 m is considered high altitude; 3500–5500 is considered very high altitude; and above 5500 m is considered extremely high altitude [3]. Numerous studies have reported that acute hypoxic responses occur when an untrained body rapidly enters an altitude of more than 2500 m [13]. Adequate acclimatization can significantly reduce the occurrence of acute hypoxic responses. It is generally recognized that adaptation is achieved by staying 6–7 days at an altitude of approximately 2000–3000 m or ascending less than 500 m per day [14]. At an altitude of 7620 m, the ambient oxygen partial pressure is 60 mmHg, and the alveolar oxygen partial pressure falls to 30 mmHg, which can cause loss of consciousness. The human body, even after acclimation, cannot stay at this altitude for a long time. We used SD rats as the model and found that most unacclimated SD rats survived for only approximately 6 h at the simulated altitude of 7620 m, and the 24 h survival rate was only 9.5%. Achieving rapid adaptation to hypoxia in extreme altitude environments (>7620 m) is a challenging problem.

WA is a chemical component extracted from the Indian herb *Withania somnifera* [4]. WA has anti-inflammatory, antidiabetic, anticancer, and antiangiogenic effects, which have attracted widespread interest in this substance worldwide. WA exerts its anticancer effect by inhibiting the ubiquitin–proteasome pathway, inhibiting cell cycle progression, regulating oxidative stress, inducing cancer cell senescence, and inhibiting NF-κB [4]. In vivo studies in mice have shown that WA has inhibitory effects on prostate cancer, breast cancer, pancreatic cancer, thyroid cancer, and ovarian cancer [4,15,16,17]. Other studies have shown that WA is a leptin sensitizer, exerting a strong antidiabetic effect in mice [18]. More recently, reports have indicated that WA can also be used as a potential drug for the treatment of coronavirus disease 2019 (COVID-19) [19].

As WA is a pleiotropic drug, we were encouraged to find that it significantly improved the hypoxia tolerance of SD rats. After intraperitoneal injection of WA for 7 days at a dosage of 2 mg/kg/d, we found that the survival of SD rats at the simulated altitude of 7620 m was significantly improved and that the 24 h survival rate increased to 85.7%. In addition, myocardial hypoxic injury was significantly reduced, and cardiac function after hypoxia was effectively improved in WA-pretreated rats. Moreover, our in vitro experiments confirmed that acute hypoxic injury was significantly reduced in cardiomyocytes pretreated with WA, as evidenced by the significant reductions in the apoptosis and necrosis rates. Previous research also confirmed that the therapeutic dose of WA has minimal toxic effects on normal tissues and cells [20,21], explaining why WA has a cytotoxic effect on cancer cells but does not damage—or even protects—normal cells. However, the reasons for these effects are still not clear.

The most fundamental cause of hypoxia-induced injury is the O_2_ deficiency-induced lack of ATP supply to cells, especially those in cardiac and brain tissues, which have a great demand for energy. Extreme O_2_ deficiency can easily cause the death of cardiac and brain tissues. Mitochondria are the energy factories of cells. Electrons carried by substrates are transferred to O_2_ in the mitochondrial electron transport chain (ETC), which is coupled to the production of ATP via a process called mitochondrial respiration or oxidative phosphorylation [22]. At 7620 m, although the oxygen partial pressure of human alveoli is reduced to 30 mmHg, the oxygen partial pressure of cellular mitochondria is still above 1 mmHg, and cells can still produce ATP through oxidative phosphorylation.

In the human body, the heart is the organ with the greatest need for energy. Almost all ATP production in the adult mammalian myocardium is via mitochondrial oxidative phosphorylation; thus, mitochondria are particularly important in myocardial tissues. Diverse molecular pathways, including mitochondrial fission and fusion, mitophagy, and mitochondrial biogenesis, are involved in the regulation of mitochondrial quality; these pathways are collectively referred to as mitochondrial quality control mechanisms [23,24]. The mitochondrial fission process in mammalian cells is regulated mainly by DRP1, which is localized in the cytoplasm. Under the action of mitochondrial fission-inducing factors, DRP1 binds to the outer mitochondrial membrane protein FIS1 to induce mitochondrial fission [25]. Mitochondrial fusion involves the fusion of the inner membrane and the outer membrane. MFN1 and MFN2 mediate mitochondrial outer membrane fusion, and OPA1 is involved mainly in mitochondrial inner membrane fusion [25]. Mitophagy can remove damaged, aged, and dysfunctional mitochondria from cells. Mitophagy is activated mainly via three receptor-mediated pathways: the PINK1/Parkin-mediated, FUNDC1-mediated, and BNIP3-mediated mitophagy pathways [26]. Studies have demonstrated that mitophagy induced under hypoxia depends mainly on the activation of BNIP3 [26,27]. PGC-1α is a transcriptional cofactor that plays a core regulatory role in mitochondrial biogenesis [28]. PGC-1α promotes the expression of TFAM by activating the downstream transcription factors NRF1 and NRF2, which can promote the transcription of mitochondrial enzyme-related genes and interact with TFAM to drive mtDNA transcription and replication [28].

This study showed that the transcriptional levels of the mitochondrial biogenesis-related genes PGC-1α, NRF2, and TFAM, as well as the levels of the encoded proteins, were significantly increased in myocardial tissues of rats pretreated with WA. The mRNA and protein levels of the mitophagy-related gene BNIP3 were also significantly increased, whereas the expression of proteins associated with mitochondrial fission and fusion did not change, suggesting that WA has an impact on mitochondrial quality control mechanisms—mainly regulating the processes of mitochondrial biogenesis and mitophagy—but has no significant effect on mitochondrial fission or fusion. Although the protein expression levels of PGC-1α, NRF2, and TFAM in myocardial tissues of the rats in the WH group did not change compared with those in the W group, the corresponding mRNA levels were significantly reduced. The results of our in vitro experiments confirmed this finding. WA pretreatment significantly enhanced mitochondrial biogenesis and BNIP3-mediated mitophagy in cardiomyocytes in the W group, and the mRNA levels of PGC-1α, NRF2, and TFAM were significantly decreased in cardiomyocytes in the WH group (after acute hypoxia) compared with those in the W group (before hypoxia). These results suggest that acute hypoxia may inhibit the process of mitochondrial biogenesis. Our previous study has verified that the BNIP3-mediated mitophagy plays an important role in clearing damaged mitochondria, further reduces the production of mitochondrial-derived ROS, and prevents further damage caused by increased ROS [29,30]. In this study, we confirmed that mitochondrial damage was significantly reduced in myocardial tissues and cells after WA pretreatment and that the production of ROS in tissues and cells was significantly reduced. Both in vivo and in vitro, qPCR analysis of mtDNA and TEM imaging showed that the H group had significantly fewer mitochondria than the Con group, while the WH group had significantly fewer mitochondria than the W group; moreover, the few remaining mitochondria in the H group were dysfunctional. Both of these results suggest a significant decrease in the number of mitochondria in myocardial tissue and cells after hypoxia. On the one hand, the decrease in the number of mitochondria after hypoxia may be due to hypoxia-induced inhibition of mitochondrial biogenesis. On the other hand, hypoxia-induced rupture and dissolution of mitochondria is also an important cause of the reduction in mitochondria. The protection role of PGC-1α in acute hypoxia was confirmed in this study. Because WA pretreatment upregulated PGC-1α-mediated mitochondrial biogenesis, even though the number of mitochondria decreased after hypoxia, the number of mitochondria in myocardial tissues and cells in the WH group was still similar to that in the Con group. Most importantly, these new mitochondria exhibited normal function. The results of mitochondrial function assays suggested that the reduction in the MMP in cardiomyocytes under acute hypoxia was alleviated after WA pretreatment and that the level of mitochondrial respiration was maintained to ensure the production of ATP under acute hypoxia.

**Figure 9:**
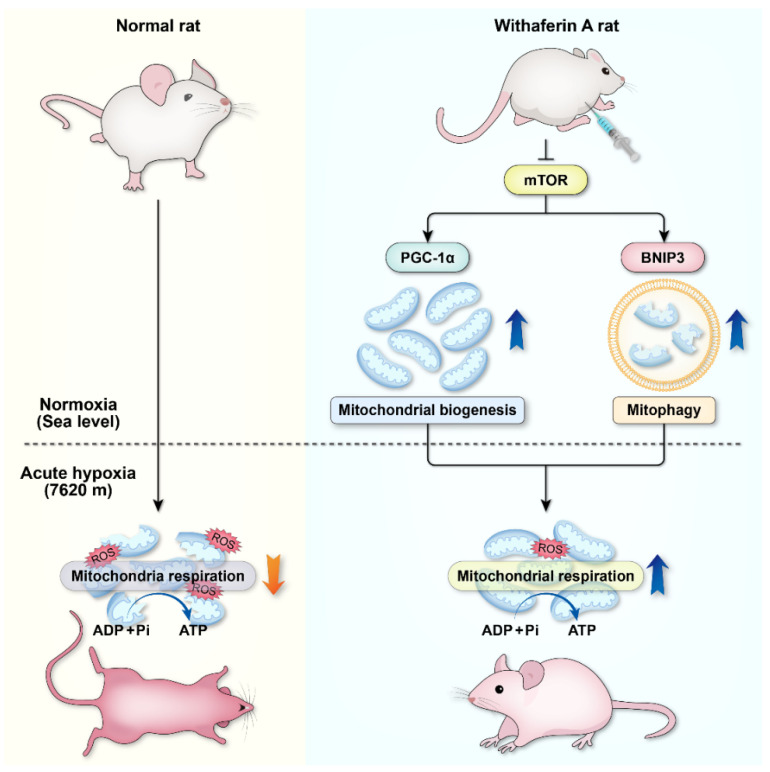
Schematic diagram illustrating that withaferin A pretreatment enhances the adaptability to extreme hypoxia by enhancing PGC-1α-mediated mitochondrial biogenesis and BNIP3-mediated mitophagy. Normal SD rats have difficulty surviving in the extreme hypoxic environment due to mitochondrial damage and insufficient ATP supply. Withaferin A pretreatment can significantly improve survival in rats in extremely hypoxic environments. Taken together with our previous study [30], the upstream regulator, mTOR, plays an important role in regulating these two pathways. By inhibiting the mTOR, on the one hand, WA enhances the BNIP3-mediated mitophagy, which eliminates hypoxia-damaged mitochondria and prevents the induction of apoptosis. On the other hand, WA promotes the PGC-1α-mediated mitochondrial biogenesis, which can supplement functional mitochondria and maintain mitochondrial respiration to ensure mitochondrial ATP production under acute extreme hypoxia. These two complementary pathways, mitophagy and mitochondrial biogenesis, improve the body’s ability to rapidly adapt to extremely hypoxic environments.

In summary, this study has demonstrated that acute hypoxia can cause severe mitochondrial damage and decrease the number of mitochondria, making it difficult to maintain effective mitochondrial respiration. WA can improve the body’s extreme hypoxia tolerance. Our previous findings highlight the protective role of mTOR in acute hypoxic adaptation, and targeted regulation of mTOR could be a new strategy to improve acute hypoxic tolerance in the body [30]. As the upstream regulator of PGC1α and BNIP3, mTOR should be an important target for WA to play its role. By inhibiting the mTOR, on the one hand, WA activates BNIP3-mediated mitophagy for rapid clearance of mitochondria damaged by extreme hypoxia, thereby preventing the increase in ROS and the activation of apoptosis caused by dysfunctional mitochondria. On the other hand, WA activates mitochondrial biogenesis, and the cleared mitochondria are continuously replaced by newly formed mitochondria, which exhibit normal mitochondrial respiratory function. Mitophagy and mitochondrial biogenesis coordinate with each other to ensure that the energy supply of the body is maintained in an extremely hypoxic environment (Figure 9). Therefore, WA can be used as an effective drug to improve the body’s adaptability to extreme hypoxia. Mitophagy and mitochondrial biogenesis play an important role in the rapid adaptation to extreme hypoxia. Targeted regulation of mitophagy or mitochondrial biogenesis could be a novel strategy to improve tolerance to extreme hypoxia.

## Data Availability

The datasets used and/or analyzed during this study are available from the corresponding author on reasonable request.

## Supplementary Materials

The following supporting information can be downloaded at: https://www.mdpi.com/article/10.3390/cells12010085/s1, Figure S1: Twenty-four hours under 7620 m survival curves for SD rats with and without treatment with WA; Figure S2: WA improved the diastolic function of hearts in rats under extreme hypoxia; Figure S3: Effect of WA on the expression of mitochondrial fusion and fission proteins in myocardium of rats; Figure S4: The effect of WA on the cardiomyocyte’s viability used with different concentration and action time; Figure S5: Overexpression of PGC-1α increased mitochondrial number and improved mitochondrial respiratory function of cardiomyocytes during acute hypoxia; Table S1: urvival rates of adult SD rats (10 ~ 12 weeks old) after exposure to 7620 m for 24 h; Table S2: Details of primary antibodies; Table S3: Details of second antibodies; Table S4: Details of mRNA primers (R, rat; H, human).
