# Supplementary material for: Withaferin A Enhances Mitochondrial Biogenesis and BNIP3-Mediated Mitophagy to Promote Rapid Adaptation to Extreme Hypoxia
Source: Cells. 2022 Dec 25;12(1):85. doi: 10.3390/cells12010085 (PMC9818179; doi:10.3390/cells12010085)
Supplement: Supplementary file 1 [file cells-12-00085-s001.zip › cells-2037095-supplementary.pdf]

Supplemental material

Withaferin A enhances mitochondrial biogenesis and BNIP3-mediated mitophagy to promote rapid adaptation to extreme hypoxia

Ruzhou Zhao[1,2*], Yixin Xu[3*], Xiaobo Wang[1], Xiang Zhou[1], Yanqi Liu[1], Shuai Jiang[1], Lin Zhang[1], Zhibin Yu[1]

Supplemental Figures S1-S3

Supplemental Table S1-S4

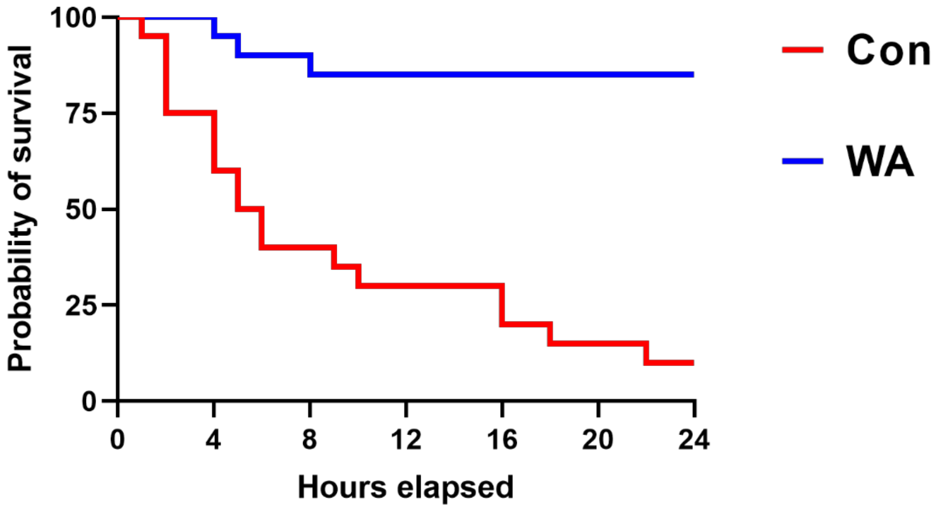

Figure S1. Twenty-four hours under 7620 m survival curves for SD rats with and without treatment with WA.

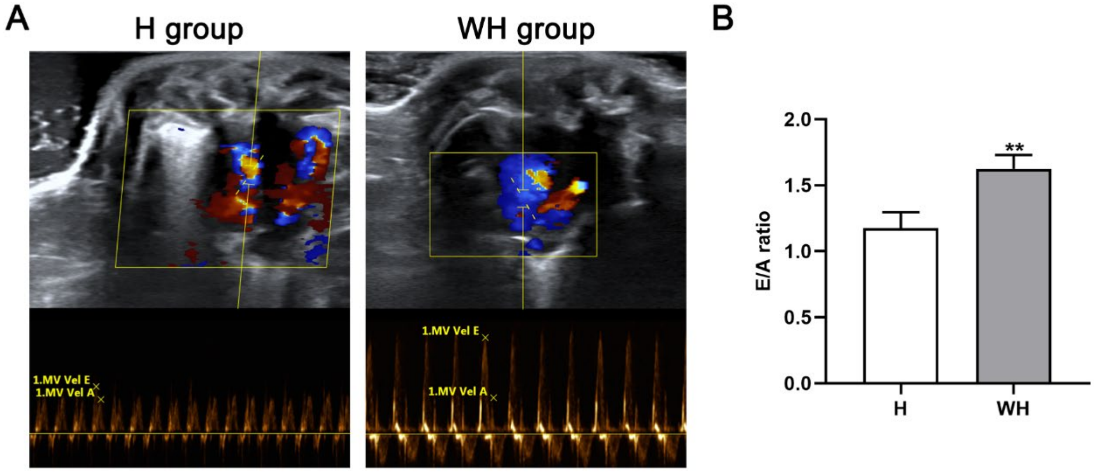

Figure S2. WA improved the diastolic function of hearts in rats under extreme hypoxia. (A) Representative images of pulse-wave Doppler. (B) Quantified results of E/A ratio. $^{**}p < 0.01$

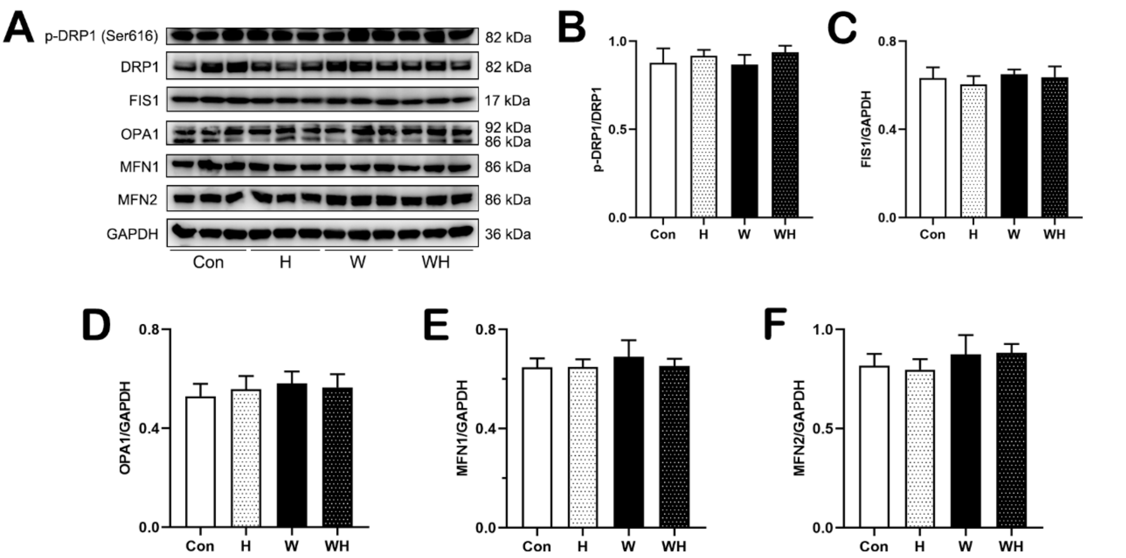

Figure S3. Effect of WA on the expression of mitochondrial fusion and fission proteins in myocardium of rats. (A) Representative immunoblots of indicated proteins. (B-F) Statistical graphs of the protein levels of p-DRP1 (B), FIS1 (C), OPA1 (D), MFN1 (E), and MFN2 (F).

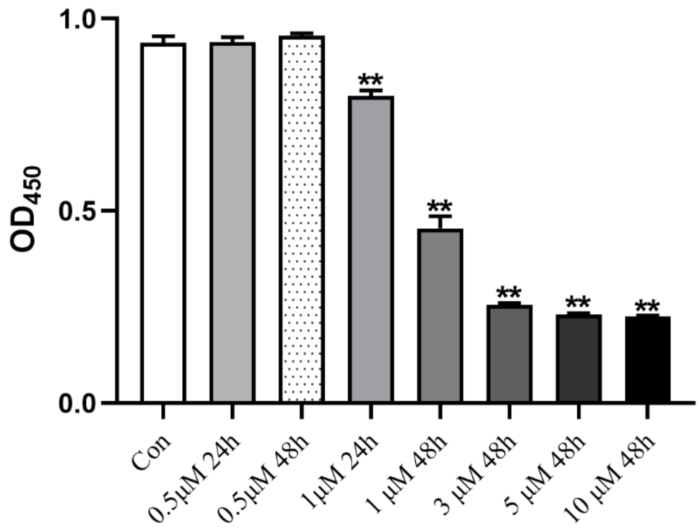

Figure S4. The effect of WA on the cardiomyocyte's viability used with different concentration and action time. $^{**}p < 0.01$.

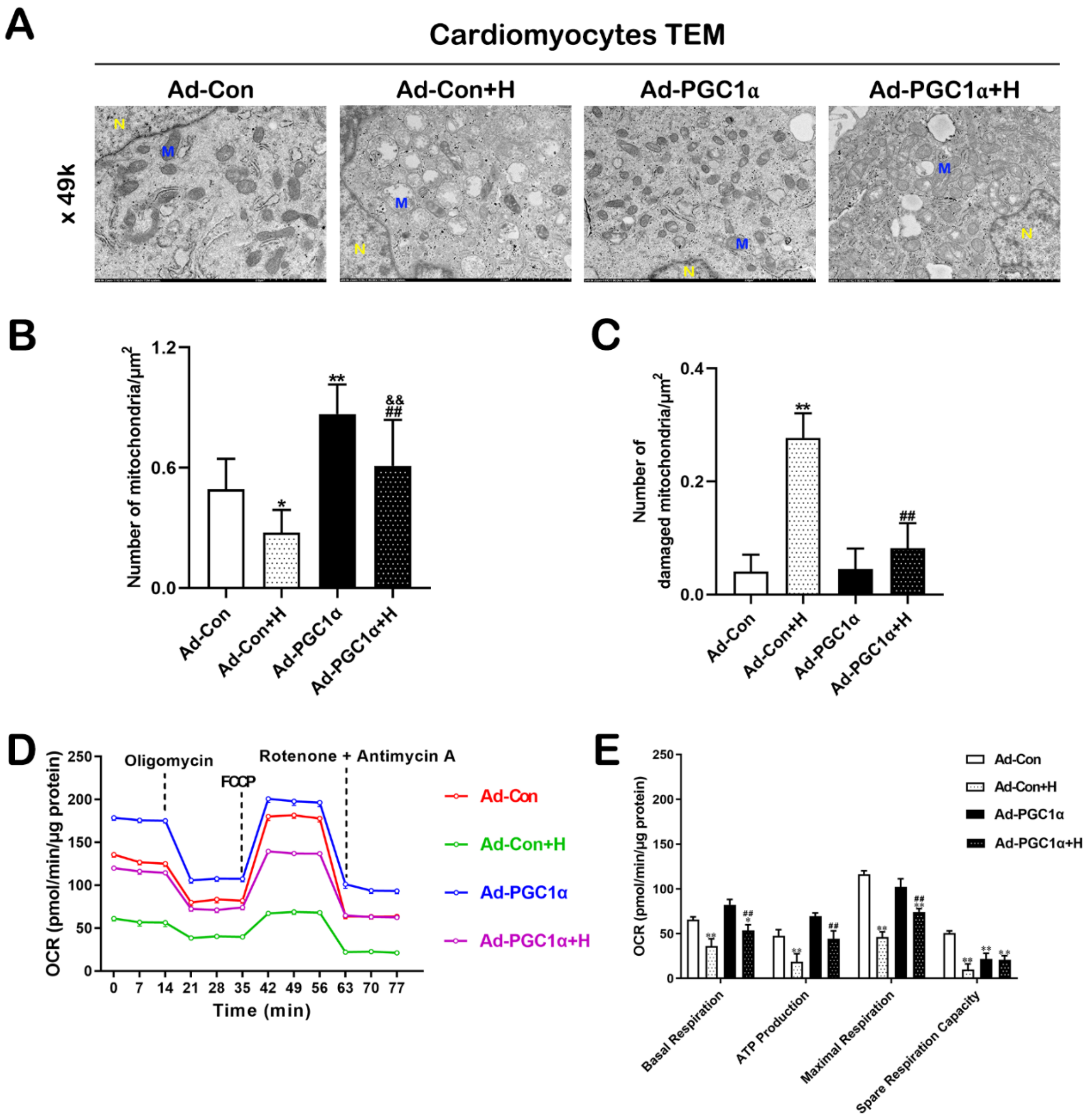

Figure S5. Overexpression of PGC-1α increased mitochondrial number and improved mitochondrial respiratory function of cardiomyocytes during acute hypoxia. (A) Representative electron micrograph of cardiomyocytes. M, mitochondria. N, nucleus. Scale bars = 2 μm. (B-C) Number of mitochondria or damaged mitochondria per μm^2. (D-E) OCR curves and quantification of related indices. n = 5. $^{**}p < 0.01$ or $^{*}p < 0.05$ vs. the Ad-Con group. $^{\#\#}p < 0.01$ vs. the Ad-Con+H group. $^{\&\&}p < 0.01$ vs. the Ad-PGC1α+H group.

Table S1. Survival rates of adult SD rats (10 ~ 12 weeks old) after exposure to 7620 m for 24 h

	T	S	R (%)
Con	21	2	9.5
WA	21	18	85.7

Con, normal SD rats; WA, SD rats i.p. with 2mg/kg/d for 7d; T, total number of SD rats in experiments; S, survival number of SD rats after exposure to 7620 m for 24 h; R, survival rate of SD rats after exposure to 7620 m for 24 h.

Table S2. Details of primary antibodies

Antibody	Manufacturer and Cat No.	Application	Dilute proportion
p-DRP1	Cell Signaling Technology, #3455	WB	1:1000
DRP1	Cell Signaling Technology, #8570	WB	1:1000
FIS1	Proteintech, #10956-1-AP	WB	1:1000
OPA1	Abcam, #ab42364	WB	1:1000
MFN1	Proteintech, #13798-1-AP	WB	1:1000
MFN2	Proteintech, #12186-1-AP	WB	1:2000
PGC-1α	Proteintech, #66369-1-Ig	WB	1:5000
NRF2	Abcam, #ab89443	WB	1:1000
NRF1	Proteintech, #12482-1-AP	WB	1:1000
TFAM	Proteintech, #22586-1-AP	WB	1:2000
Beclin1	Proteintech, #11306-1-AP	WB	1:1000
P62	Proteintech, #18420-1-AP	WB	1:1000
LC3	Abcam, #ab48394	WB	1:1000
BNIP3	Abcam, #ab109362	WB	1:1000

GAPDH	Proteintech, #60004-1-Ig	WB	1:5000

Table S3. Details of second antibodies

Antibody	Manufacturer and Cat No.	Application	Dilute proportion
Anti-rabbit, HRP-linked antibody	Cell Signaling Technology, #7074	WB	1:5000
Anti-mouse, HRP-linked antibody	Cell Signaling Technology, #7076	WB	1:5000

Table S4. Details of mRNA primers (R, rat; H, human)

Genes/Origin		Forward primer	Reverse primer
β-actin (mRNA)	R	CCCATCTATGAGGGTTACGC	TTTAATGTCACGCACGATTTC
	H	GCACTCTTCCAGCCTTCCTTCC	GCGGATGTCCACGTCACACTTC
PGC-1α	R	CCACTACAGACACCGCACACATC	GTATTCGTCCCTCTTGAGCCTTTCG
	H	TCCAGGTCAAGATCAAGGTCTCCAG	GTGCGGTGTCTGTAGTGGCTTG
NRF2	R	GACAAACATTCAAGCCGATTAG	TTATTCTTCCCTCTCCTGCG
	H	AGTCCAGAAGCCAAACTGACAGAAG	GGAGAGGATGCTGCTGAAGGAATC
NRF1	R	TCTGCTGTGGCTGATGGAGAGG	GATGCTTGCGTCGTCTGGATGG
	H	AATTATTCTGCCGTGGCTGATGGAG	CCTCTGATGCTTGCGTCGTCTG
TFAM	R	GTGATCTCATCCGTCGCAGTGTG	TGCCCAATCCCAATGACAACTCTG
	H	TGGCGTTTCTCCGAAGCATGTG	TGCCAAGACAGATGAAAACCACCTC
BNIP3	R	TCTTCTTGCTTGCAGGATGAGGATTC	AGCAGAGAGATAAAGGCGTAACACAAC
	H	AGCATGAGTCTGGACGGAGTAGC	TCTGTTGGTATCTTGTGGTGTCTGC
β-actin (DNA)	R	TCGTGCGTGACATTAAAGAG	ATTGCCGATAGTGATGACCT
	H	TCGTGCGTGACATTAAGGAGAAGC	TGAGAGGGAAATGAGGGCAGGAC
ND-1	R	AAGCGGCTCCTTCTCCCTACAAAT	GAAGGGAGCTCGATTTGTTTCTGC
	H	TCAAACTCAAACTACGCCCTGATCG	GTTCGGTTGGTCTCTGCTAGTGTG
